# Survey of the rubber tree genome reveals a high number of cysteine protease-encoding genes homologous to *Arabidopsis SAG12*

**DOI:** 10.1371/journal.pone.0171725

**Published:** 2017-02-06

**Authors:** Zhi Zou, Jianting Liu, Lifu Yang, Guishui Xie

**Affiliations:** 1 Danzhou Investigation & Experiment Station of Tropical Crops, Ministry of Agriculture, Rubber Research Institute, Chinese Academy of Tropical Agricultural Sciences, Danzhou, Hainan, P. R. China; 2 Crops Research Institute, Fujian Academy of Agricultural Sciences, Fuzhou, Fujian, P. R. China; Universidade Federal de Vicosa, BRAZIL

## Abstract

*Arabidopsis thaliana SAG12*, a senescence-specific gene encoding a cysteine protease, is widely used as a molecular marker for the study of leaf senescence. To date, its potential orthologues have been isolated from several plant species such as *Brassica napus* and *Nicotiana tabacum*. However, little information is available in rubber tree (*Hevea brasiliensis*), a rubber-producing plant of the Euphorbiaceae family. This study presents the identification of *SAG12*-like genes from the rubber tree genome. Results showed that an unexpected high number of 17 rubber orthologues with a single intron were found, contrasting the single copy with two introns in *Arabidopsis*. The gene expansion was also observed in another two Euphorbiaceae plants, castor bean (*Ricinus communis*) and physic nut (*Jatropha curcas*), both of which contain 8 orthologues. In accordance with no occurrence of recent whole-genome duplication (WGD) events, most duplicates in castor and physic nut were resulted from tandem duplications. In contrast, the duplicated *HbSAG12H* genes were derived from tandem duplications as well as the recent WGD. Expression analysis showed that most *HbSAG12H* genes were lowly expressed in examined tissues except for root and male flower. Furthermore, *HbSAG12H1* exhibits a strictly senescence-associated expression pattern in rubber tree leaves, and thus can be used as a marker gene for the study of senescence mechanism in *Hevea*.

## Introduction

Leaf senescence, the last stage of leaf development, is a complex but highly regulated developmental process that is controlled by an array of internal and external factors [1,2]. Internal factors include age and levels of plant hormones, whereas external factors include shade/darkness, desiccation, drought, heat, nutrient stresses, pathogen infection and various hormones such as cytokinin (CK), auxin, ethylene (ET), jasmonic acid (JA), salicylic acid (SA) and abscisic acid (ABA) [3,4]. These factors trigger a series of coordinated events such as shifts in gene expression, loss of chlorophyll, reduction of photosynthesis, degradation of macromolecules, relocation of nutrients, the breakdown of organelles, and finally, cell death [5,6]. In the senescent leaves typical with a yellow color [7], protein degradation is one of the most important hydrolytic processes and many SAGs (senescence-associated genes) encoding proteases are synthesized *de novo* or induced [8]. *SAG12*, a well-known *Arabidopsis* gene encoding a papain-like cysteine protease, exhibits a strictly senescence-associated expression pattern in leaves and flowers [9–11]. *AtSAG12* is widely used as a molecular marker for the study of developmental senescence, since its expression is developmentally controlled and cannot be induced by various stresses (e.g. desiccation, dark incubation and wounding) or hormones (e.g. ABA and ET) in young, detached *Arabidopsis* leaves [11]. However, in mature leaves that are ready for senescence, it can be induced by detachment, ABA and ET [12]. Although the exact physiological role of AtSAG12 is still not known, its colocalization with Rubisco and other stromal proteins in SAVs (senescence-associated vacuoles) in *Arabidopsis* was observed [13]. Moreover, studies indicated that the *AtSAG12* promoter is the target of transcription factors *AtWRKY53* (a senescence inducer) and *AtWRKY57* (a senescence inhibitor) [14,15], and the promoter has been fused to IPT to form an autoregulatory senescence inhibition system widely applied to plant species [16,17]. To date, the potential orthologues of AtSAG12 were isolated from several species, e.g., *BnSAG12-1* and *BnSAG12-2* in *Brassica napus* [18], *SPG31* in *Ipomoea batatas* [19], *ccyp* in *Gossypium hirsutum* [20], *NtCP1* and *NtSAG12* in *Nicotiana tabacum* [21,22].

Para rubber tree (*Hevea brasiliensis* Muell. Arg., *2n* = 36), a perennial big tree of the Euphorbiaceae family, is economically important for the production of natural rubber (*cis*-1,4-polyisoprene), an essential industrial raw material [23,24]. Although native to the Amazon basin, the increasing demand of natural rubber has prompted the commercial cultivation of rubber trees in the tropical regions of Asia, Africa, and Latin America [25]. Natural rubber is specifically synthesized and stored in the highly differentiated vessels termed laticifers, which are located in the soft inner bark of the tree trunk and periodically differentiated from the vascular cambium [26]. Upon tapping, the laticifer cytoplasm is expelled in the form of latex due to the high turgor pressure inside [27,28]. Over the past decades, the latex yield has been significantly increased for the wide cultivation of high-yielding clones and the extensive utilization of ethephon (an ET generator) [29,30]. Meanwhile, steadily increased occurrence of a complex physiological syndrome called TPD (Tapping Panel Dryness), which is characterized as tapping incision blocked partly or entirely during latex exploiting, cause great losses of latex production [30,31]. Although a great deal of effort has been made on TPD, the molecular mechanism underlying remains poorly understood. Nuclear DNA fragmentation, upregulation of a high number of senescence-associated genes and downregulation of many anti-apoptosis-associated genes in TPD-affected trees [32–36] suggest that TPD is a type of programmed cell death (PCD), probably due to overproduction of reactive oxygen species (ROS) resulted from high-strength tapping and ET over-stimulating [37]. Nevertheless, the functional characterization of TPD-associated genes is hindered for several reasons: rubber tree is a perennial plant species with long breeding time and long juvenile phase of 7–8 years before tapping, where the genetic transformation system is still not well established yet [25]; the induced TPD is destructive on rubber production; and, no laticifer can be found in model plants such as *Arabidopsis* and tobacco [33]. As a type of PCD, TPD is more likely to share some features of leaf senescence. Thereby, the information about rubber tree leaf senescence may improve our knowledge on TPD.

To explore molecular markers for the study of senescence mechanism in rubber tree, the present study took advantage of the recently available genome sequences [38] to identify the potential orthologues of AtSAG12. Furthermore, their evolutionary pattern and expression profiles over various tissues including natural and ET-induced senescent leaves were also investigated.

## Materials and methods

### Identification of *SAG12*-like genes in the rubber tree genome

All the deduced protein sequences of *Arabidopsis* were downloaded from TAIR10 [39]. To identify the orthologues of *Arabidopsis* SAG12 in rubber tree, a two-step approach was used. First, the amino acid sequence of AtSAG12 (TAIR10 accession number AT5G45890) was used as the query to search the Reyan7-33-97 genome [38], and sequences with an E-value of less than 1e^-5^ in the TBLASTN search [40] were collected; the positive genomic sequences were predicted using GeneMark.hmm [41], and the gene models were further validated with cDNAs, expressed sequence tags (ESTs) and RNA sequencing reads when available. Homology search for nucleotides or ESTs was performed using BLASTN, and sequences with the identity of more than 98% were taken into account. RNA sequencing data available in NCBI SRA (http://www.ncbi.nlm.nih.gov/) were also used for the expression annotation: the reads were first filtered by removing adaptor sequences, adaptor-only reads and low quality reads containing more than 50% bases with Q-value≤5; read alignment was performed using Bowtie 2 [42], and mapped read number of more than one was counted as expressed. Unless specific statements, the tools used in this study were performed with default parameters.

Subsequently, the deduced amino acid sequences of putative homologues were used as the queries to search the local *Arabidopsis* proteome database; if the best hit in the BLASTP search was AtSAG12, the gene was defined as the true orthologue of AtSAG12. Tandem or proximal duplications were considered when two duplicated genes were consecutive in the genome and separated by 20 or fewer gene loci, respectively.

### Identification of *SAG12*-like genes in another two Euphorbiaceae plants

Rubber tree is a diploid plant that was shown to have undergone a recent whole-genome duplication (WGD) event [38,43]. To investigate the recent evolutionary pattern of rubber tree SAG12 homologues, a similar approach as described above was adopted to identify orthologues from the genomes of another two Euphorbiaceae plants, castor bean (*Ricinus communis* L.) and physic nut (*Jatropha curcas* L.). Both castor and physic nut underwent no recent WGD [44,45], and their genome sequences were downloaded from Phytozome v11 [46] and NCBI GenBank, respectively.

### Sequence alignments and phylogenetic analysis

Multiple alignments were performed using MUSCLE [47]. The alignment was displayed using BoxShade (http://www.ch.embnet.org/software/BOX_form.html). The unrooted phylogenetic tree was constructed using MEGA 6.0 [48] with the maximum likelihood method and with the bootstrap test replicated 1000 times.

### Protein properties and conserved motif analysis

Protein properties such as the molecular weight (MW), isoelectric point (*p*I), and grand average of hydropathicity (GRAVY) were calculated using ProtParam (http://web.expasy.org/protparam/). The protein subcellular localization was predicted using iPSORT [49], and the location of signal peptide cleavage site was predicted using SignalP 4 [50]. Analysis for conserved motifs in proteins was performed using MEME [51]. The optimized parameters were: any number of repetitions; maximum number of motifs, 15; and the optimum width of each motif, between 6 and 50 residues. And the MAST program was used to search detected motifs in protein databases.

### Plant materials, RNA isolation and cDNA synthesis

*In vitro* plantlets of clone Reyan7-33-97 used in this study were obtained *via* secondary somatic embryogenesis [52], and the bagged plantlets were grown in a greenhouse illuminated with natural sunlight. Various tissue samples such as root, bark, laticifer, xylem, shoot apex, leaf and petiole were collected and subjected to total RNA extraction as described before [53]. The leaf tissue included leaves of different developmental stages such as bronze, color-change, pale-green, mature, early-senescent and mid-senescent, where the early- and mid-senescent leaves were defined by the chlorophyll content of 75–85% and 45–55% relative to mature leaves, respectively. The mature leaves were also treated with 50 μM ethephon to induce senescence *in vitro*, and the induced senescent leaves were collected when the chlorophyll content was dropped to 45–55%.

The isolated RNA was subsequently treated with RNase-Free DNase I (Takara), and the first-strand cDNA was synthesized from 2 μg of total RNA with M-MLV reverse transcriptase (Promega) according to the manufacturer's instructions.

### Gene expression analysis

Along with the genome sequencing, we have also sequenced several tissue transcriptomes of Reyan7-33-97 with the Illumina platform, i.e., root (NCBI SRA accession number SRX1554786), leaf (SRX1554799), bark (SRX1554797), laticifer (SRX1554800), female flower (SRX1554813), male flower (SRX1554814) and seed (SRX1554817) [38]. Thereby, the relative expression levels of *HbSAG12H* genes in these tissues were first examined: the filtered reads were mapped to the coding sequences (CDS) of identified *HbSAG12H* genes using Bowtie 2 [42], and the FPKM (fragments per kilobase of exon per million fragments mapped) method [54] was adopted for the quantification.

To identify *HbSAG12H* genes expressed in senescent leaves, 17 primer pairs (see **S1 Table**) were designed according to the genome sequences, and the RT-PCR (reverse transcriptase polymerase chain reaction) was performed to amplify the target CDS by using the synthesized cDNAs as the template. The PCR products were cloned into the pMD18-T vector (Takara) and sequenced with an ABI3730xl DNA Analyzer (Life Technologies). Semi-quantitative RT-PCR analysis was performed with gene-specific primers **(**SAG12H1F: 5’ AAC CCT TTG TCG TCC TCT GG 3'; SAG12H1R: 5’ TTT GCT TCT CGT CTG CGT CT 3'**)**, and the *Hb18S rRNA* [53] was used as the internal control. The PCR conditions were listed in **S1 File**, and at least three replicates were performed for each sample of three biological replicates. A 10 μL sample of the PCR products was analyzed by electrophoresis on 1.5% agarose gel containing ethidium bromide.

## Results

### Characterization of 17 *SAG12*-like genes in rubber tree

The initiative BLAST search resulted in 54 loci putatively encoding *Arabidopsis* SAG12 homologues from the rubber tree genome, and 17 out of them were proved to be true orthologues by the reciprocal BLASTP. These orthologues were denoted as *HbSAG12H1*–*17*, which were found to be distributed across 9 out of 7,453 scaffolds [38], i.e., scaffold0048 (5), scaffold0247 (2), scaffold0583 (2), scaffold0696 (2), scaffold1445 (1), scaffold2360 (2), scaffold0683 (1), scaffold0420 (1) and scaffold1086 (1) (**Table 1**). Interesting, a high number of *HbSAG12H* genes are organized in cluster, and the CDS of these genes exhibit relatively high identity, e.g., 97.8% between *HbSAG12H2* and *HbSAG12H3*, 80.2% between *HbSAG12H6* and *HbSAG12H7*, 96.0% between *HbSAG12H8* and *HbSAG12H9*, 99.8% between *HbSAG12H10* and *HbSAG12H11*, 78.2% between *HbSAG12H13* and *HbSAG12H14*, 80.0% between *HbSAG12H15* and *HbSAG12H16*.

**Table 1:** List of *HbSAG12H* genes identified in this study.

Gene name	Scaffold	Identified position	Nucleotide length (bp, from start to stop codons)		Intron NO.	EST hits	Examined tissues								AA	MW (Da)	*p*I	GRAVY	iPSORT^9^	SignalP^10^
			CDS	Gene			Shoot apex^1^	Leaf^2^	Laticifer^3^	Bark^4^	Root^5^	Flower^6^	Seed^7^	Somatic embryogenesis^8^						
*HbSAG12H1*	scaffold0683	458159–455326	1038	2215	1	0	ND	Yes	ND	ND	Yes	Yes	ND	ND	345	38160.0	7.99	-0.440	S	26 and 27
*HbSAG12H2*	scaffold0696	270814–272206	1023	1113	1	0	ND	Yes	ND	ND	Yes	ND	ND	ND	340	37352.7	5.24	-0.458	S	27 and 28
*HbSAG12H3*	scaffold0696	267181–268628	1023	1113	1	0	ND	ND	ND	ND	Yes	ND	ND	ND	340	37413.8	5.09	-0.446	S	27 and 28
*HbSAG12H4*	scaffold1445	17656–16340	1023	1111	1	0	ND	Yes	ND	Yes	Yes	ND	ND	ND	340	37591.1	5.18	-0.460	S	27 and 28
*HbSAG12H5*	scaffold0048	1332422–1333546	1032	1125	1	0	ND	ND	ND	ND	ND	Yes	Yes	ND	343	38172.3	8.00	-0.248	S	26 and 27
*HbSAG12H6*	scaffold0048	1334269–1335579	1026	1199	1	0	ND	ND	ND	ND	Yes	ND	Yes	ND	341	38041.6	5.25	-0.485	S	24 and 25
*HbSAG12H7*	scaffold0048	1352389–1353536	1020	1148	1	0	ND	ND	ND	ND	ND	ND	Yes	ND	339	38151.5	9.10	-0.438	S	26 and 27
*HbSAG12H8*	scaffold0048	1327811–1329006	1044	1174	1	0	ND	ND	ND	ND	ND	Yes	ND	ND	347	38578.8	8.85	-0.423	S	25 and 26
*HbSAG12H9*	scaffold0048	1282306–1283479	1044	1174	1	0	ND	ND	ND	ND	ND	Yes	ND	ND	347	38436.4	8.43	-0.443	S	25 and 26
*HbSAG12H10*	scaffold2360	12544–13717	1044	1174	1	0	ND	ND	ND	ND	ND	Yes	ND	ND	347	38452.4	8.24	-0.426	S	25 and 26
*HbSAG12H11*	scaffold2360	24160–25333	1044	1174	1	0	ND	ND	ND	ND	ND	Yes	ND	ND	347	38484.5	8.43	-0.432	S	25 and 26
*HbSAG12H12*	scaffold0420	383321–384587	1029	1282	1	0	ND	ND	ND	Yes	ND	Yes	ND	ND	342	38078.1	8.60	-0.430	S	24 and 25
*HbSAG12H13*	scaffold0247	27510–26368	1032	1134	1	0	ND	ND	ND	ND	Yes	Yes	ND	ND	343	38107.7	4.90	-0.300	S	26 and 27
*HbSAG12H14*	scaffold0247	29905–28558	1029	1136	1	0	Yes	Yes	ND	Yes	Yes	ND	ND	Yes	342	37452.8	5.03	-0.374	S	26 and 27
*HbSAG12H15*	scaffold0583	111976–113311	1029	1112	1	0	ND	Yes	ND	Yes	Yes	Yes	Yes	ND	342	37592.6	4.65	-0.463	S	26 and 27
*HbSAG12H16*	scaffold0583	114772–116144	1029	1138	1	0	ND	ND	ND	ND	Yes	ND	Yes	ND	342	37610.8	4.72	-0.383	S	26 and 27
*HbSAG12H17*	scaffold1086	195937–194788	1029	1252	1	0	ND	Yes	Yes	Yes	Yes	Yes	Yes	ND	342	37505.7	4.80	-0.454	S	26 and 27

^1^ Based on the 454 transcriptome data under the NCBI SRA accession number of DRX000223.

^2^ Based on the 454 transcriptome data of SRX451708 and Illumina transcriptome data of SRX206128, SRX206129, SRX206130, SRX203083, SRX203085, SRX203117, SRX203118, SRX278515 and SRX1554799.

^3^ Based on the 454 transcriptome data of SRX451705 and Illumina transcriptome data of SRX037405, SRX206131, SRX206132, SRX278514, SRX1554800, SRX1554821, SRX1554824, SRX1554825 and SRX1554828.

^4^ Based on the 454 transcriptome data of SRX451707 and Illumina transcriptome data of SRX278513, SRX371361 and SRX1554797.

^5^ Based on the 454 transcriptome data of SRX451710 and Illumina transcriptome data of SRX1554786.

^6^ Based on the Illumina transcriptome data of SRX1554813 and SRX1554814.

^7^ Based on the Illumina transcriptome data of SRX1554817.

^8^ Based on the 454 transcriptome data of SRX451709.

^9^ “S” represents the signal peptide predicted by iPSORT.

^10^ The location of signal peptide cleavage site predicted by SignalP.

“Yes” represents genes expressed. “ND” represents genes undetected.

Although no corresponding ESTs can be found in GenBank (as of July 2016) for all *HbSAG12H* genes, read alignment supported their expression in at least one of the examined tissues, i.e., shoot apex, leaf, laticifer, bark, root, flower, seed and somatic embryogenesis [38,55–59] (**Table 1**). The cDNAs of *HbSAG12H1–4*, *HbSAG12H6*, *HbSAG12H13–17*, can also been isolated from roots or senescent leaves with gene-specific primers *via* RT-PCR (data not shown). Without any exception, all *HbSAG12H* genes were shown to contain a single intron. Compared with the similar length of CDS (1020–1044 bp), the gene size (from start to stop codons) of *HbSAG12H* genes is highly distinct (1111–2215 bp). The average length of the intron is about 190 bp, with the minimum of 83 bp in *HbSAG12H15* and the maximum of 1177 bp in *HbSAG12H1* (**Table 1**).

### Characterization of *SAG12*-like genes in castor and physic nut

The homology search resulted in 8 *RcSAG12Hs* and 8 *JcSAG12Hs* from the genomic sequences of castor and physic nut, respectively. The expression of these genes were all supported by available RNA sequencing reads, and a single intron was observed as that in rubber tree **(Tables 2 and 3)**.

**Table 2:** List of *JcSAG12H* genes identified in this study.

Gene name	Locus ID	Scaffold	Predicted position	Identified position	Nucleotide length (bp, from start to stop codons)		Intron NO.	EST hits	Examined tissues			AA	MW (Da)	*p*I	GRAVY	iPSORT^4^	SignalP^5^
					CDS	Gene			Leaf^1^	Root^2^	Seed^3^						
*JcSAG12H1*	JCGZ_09604	scaffold26	600732–601907	602051–600546	1038	1400	1	0	Yes	Yes	Yes	345	38263.2	7.99	-0.438	S	22 and 23
*JcSAG12H2*	JCGZ_21557	scaffold684	2264217–2264672	2264714–2271901	1038	1274	1	0	Yes	Yes	Yes	345	38403.5	8.56	-0.377	S	22 and 23
*JcSAG12H3*	JCGZ_24483	scaffold84	187704–188806	189021–187389	1020	1103	1	0	Yes	Yes	Yes	339	37598.3	5.94	-0.424	S	27 and 28
*JcSAG12H4*	JCGZ_17185	scaffold5	199542–200754	199432–200754	1023	1213	1	0	Yes	Yes	Yes	340	37417.8	5.13	-0.413	S	27 and 28
*JcSAG12H5*	JCGZ_25372	scaffold872	472278–473548	469319–470468	1023	1271	1	0	Yes	Yes	Yes	340	37276.9	6.90	-0.426	S	27 and 28
*JcSAG12H6*	JCGZ_25371	scaffold872	469319–470468	472278–473548	1023	1150	1	0	Yes	Yes	Yes	340	37592.1	5.01	-0.424	S	27 and 28
*JcSAG12H7*	JCGZ_21549	scaffold684	2211946–2213074	2213128–2211946	1035	1183	1	0	Yes	Yes	Yes	344	37978.3	4.73	-0.372	S	26 and 27
*JcSAG12H8*	-	scaffold684	-	101979–103278	1029	1128	1	0	Yes	Yes	Yes	342	37477.9	4.97	-0.413	S	26 and 27
JcSAG12H*	JCGZ_05109	scaffold170	34760–35550	34379–35679	-	-	-	-	ND	ND	ND	-	-	-	-	-	-
JcSAG12H*	-	scaffold170	-	101979–103278	-	-	-	-	ND	ND	ND	-	-	-	-	-	-
JcSAG12H*	-	scaffold170	-	123338–124277	-	-	-	-	ND	ND	ND	-	-	-	-	-	-

^1^ Based on the 454 transcriptome data under the NCBI SRA accession number of SRX020243 and Illumina transcriptome data of SRX750580, SRX1097498 and SRX997124.

^2^ Based on the Illumina transcriptome data of SRX750579.

^3^ Based on the 454 transcriptome data of SRX011411 and Illumina transcriptome data of SRX750581.

^4^ “S” represents the signal peptide predicted by iPSORT.

^5^ The location of signal peptide cleavage site predicted by SignalP.

“Yes” represents genes expressed. “ND” represents genes undetected.

“*” represents pseudogenes.

**Table 3:** List of *RcSAG12H* genes identified in this study.

Gene name	Locus ID	Scaffold	Predicted position	Identified position	Nucleotide length (bp, from start to stop codons)		Intron NO.	EST hits	Examined tissues				AA	MW (Da)	*p*I	GRAVY	iPSORT^5^	SignalP^6^
					CDS	Gene			Leaf^1^	Flower^2^	Endosperm^3^	Seed^4^						
*RcSAG12H1*	30131.t000408	scaffold30131	2504598–2505766	2504200–2506430	1089	1169	1	0	Yes	Yes	Yes	Yes	362	41112.5	6.17	-0.412	S	27 and 28
*RcSAG12H2*	28962.t000017	scaffold28962	92991–94101	92944–94101	1023	1111	1	0	Yes	Yes	Yes	ND	340	37196.5	5.22	-0.441	S	27 and 28
*RcSAG12H3*	28962.t000018	scaffold28962	96412–97522	96181–97735	1023	1111	1	0	Yes	Yes	Yes	Yes	340	37460.9	5.16	-0.424	S	27 and 28
*RcSAG12H4*	29646.t000033	scaffold29646	207629–208856	207526–209015	1050	1228	1	0	Yes	Yes	Yes	ND	349	38552.2	9.33	-0.347	S	27 and 28
*RcSAG12H5*	29646.t000034	scaffold29646	211653–212894	211496–213036	1029	1221	1	0	ND	Yes	ND	ND	342	38064.1	8.59	-0.417	S	27 and 28
*RcSAG12H6*	29900.t000065	scaffold29900	407069–405926	407069–405639	1035	1144	1	0	ND	Yes	ND	ND	344	38115.5	5.13	-0.428	S	26 and 27
*RcSAG12H7*	29910.t000015	scaffold29910	208698–206791	208876–206709	1026	1908	1	0	ND	Yes	Yes	Yes	341	37411.6	4.86	-0.458	S	26 and 27
*RcSAG12H8*	29910.t000014	scaffold29910	204533–202640	204533–202640	1029	1894	1	0	ND	Yes	Yes	Yes	342	37397.5	4.71	-0.439	S	26 and 27
RcSAG12H*	29900.t000066	scaffold29900	414779–414008	208698–206791	-	-	-	-	-	-	-	-	-	-	-	-	-	-

^1^ Based on Illumina transcriptome data under the NCBI SRA accession number of ERX021378.

^2^ Based on the Illumina transcriptome data of ERX021379.

^3^ Based on the 454 transcriptome data of SRX007402–SRX007408 and Illumina transcriptome data of ERX021375 and ERX021376.

^4^ Based on the Illumina transcriptome data of ERX021377 and SRX485027.

^5^ “S” represents the signal peptide predicted by iPSORT.

^6^ The location of signal peptide cleavage site predicted by SignalP.

“Yes” represents genes expressed. “ND” represents genes undetected.

“*” represents pseudogenes.

In physic nut, the identified *JcSAG12H* genes were shown to be distributed across 5 out of 6,023 scaffolds [44], i.e., scaffold26 (1), scaffold684 (3), scaffold84 (1), scaffold5 (1) and scaffold872 (2) (**Table 2**). Based on the linkage map containing 1208 markers [44], these scaffolds can be further anchored to 4 chromosomes, i.e., LG3 (scaffold26 and scaffold684), LG2 (scaffold84), LG4 (scaffold5) and LG9 (scaffold872). Compared with the automatic annotation, one more locus (denoted as *JcSAG12H8*) was predicted from scaffold684 (see **S2 File**). In addition, three pseudogenes, i.e., JCGZ_05109 and another two loci, were also identified from scaffold170. Three gene pairs exhibit high levels of homology, i.e., 97.8% between *JcSAG12H1* (JCGZ_09604) and *JcSAG12H2* (JCGZ_21557), 88.6% between *JcSAG12H5* (JCGZ_25372) and *JcSAG12H6* (JCGZ_25371) and 76.4% between *JcSAG12H7* (JCGZ_21549) and *JcSAG12H8*. *JcSAG12H5*/*6* and *JcSAG12H7*/*8* can be defined as tandem duplications, whereas *JcSAG12H1* and *JcSAG12H2* were defined as proximal duplications for their distribution on two distinct scaffolds of chromosome 3. Interesting, 3 out of the 7 annotated gene models were proved to be inaccurate. The locus JCGZ_09604 (*JcSAG12H1*) was predicted to encode 311 residues, however, read alignment indicated that it represents only the 3’ sequence of the gene which is promised to encode 345 residues (see **S3 File**). The locus JCGZ_21557 (*JcSAG12H2*) was predicted to encode 155 residues, however, read alignment indicated that it represents only the 5’ sequence of the gene which is promised to encode 345 residues (see **S4 File**). The locus JCGZ_21549 (*JcSAG12H7*) was predicted to encode 324 residues, however, read alignment indicated that it represents only the 3’ sequence of the gene which is promised to encode 344 residues (see **S5 File**).

In castor, 8 *RcSAG12H* genes are also distributed across 5 out of 25,878 scaffolds [43], i.e., scaffold30131 (1), scaffold28962 (2), scaffold29646 (2), scaffold29900 (1) and scaffold29910 (2) (**Table 3**). These genes on the same scaffold were defined as tandem duplications for their close organization and high sequence identity, i.e., 97.4% between *RcSAG12H7* (29910.t000015) and *RcSAG12H8* (29910.t000014), 96.8% between *RcSAG12H2* (28962.t000017) and *RcSAG12H3* (28962.t000018), and 87.5% between *RcSAG12H4* (29646.t000033) and *RcSAG12H5* (29646.t000034). Compared with *RcSAG12H4*, the CDS length of *RcSAG12H5* is 21-bp shorter which was resulted from a C/T mutation at the 3’ terminus, and this gives rise to relatively low identity between these two genes. In addition, a pseudogene (29900.t000066) derived from *RcSAG12H6* (29900.t000065) *via* tandem duplication was also found. Except for *RcSAG12H8*, the transcription regions of other seven genes were successfully extended based on read alignment. It's worth noting that the CDS length of *RcSAG12H1* is considerably longer than other orthologues in castor as well as that in physic nut and rubber tree, which was shown to be resulted from fragment loss of its 3’ terminus.

### Phylogenetic analysis of HbSAG12Hs

**Fig 1:**
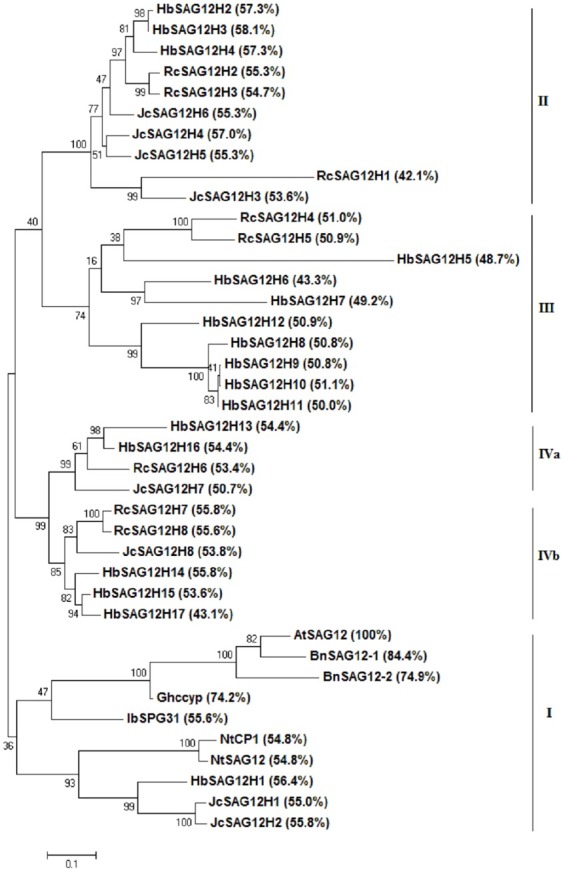
Phylogenetic analysis of SAG12 orthologues. Sequence alignment was performed using MUSCLE and the phylogenetic tree was constructed using bootstrap maximum likelihood tree (1000 replicates) method of the MEGA6 software. The distance scale denotes the number of amino acid substitutions per site, and the sequence identity of each orthologue to AtSAG12 is indicated in brackets. Species and Genbank accession numbers of reported SAG12 orthologues are as follows: BnSAG12-1 (AAD53011, *Brassica napus*), BnSAG12-2 (AAD53012, *Brassica napus*), Ghccyp (AAT34987, *Gossypium hirsutum*), NtCP1 (AAW78661, *Nicotiana tabacum*), NtSAG12 (ADV41672, *Nicotiana tabacum*) and IbSPG31 (AAK48495, *Ipomoea batatas*).

To reveal the evolutionary relationships of rubber SAG12 orthologues, an unrooted phylogenetic tree was constructed using MEGA6 from 17 HbSAG12Hs together with AtSAG12, 8 RcSAG12Hs, 8 JcSAG12Hs and orthologues reported in other plant species. As shown in **Fig 1**, these SAG12 orthologues exhibit the identity of 42.1% (RcSAG12H1) to 84.4% (BnSAG12-1) with AtSAG12, and were split into four main groups containing members from at least two examined species. Group I includes HbSAG12H1, JcSAG12H1–2, AtSAG12 and other reported orthologues. HbSAG12H1 and two JcSAG12Hs were clustered together, showing closer relationship with two *Nicotiana* orthologues than AtSAG12. Group II includes HbSAG12H2–4, JcSAG12H3–6 and RcSAG12H1–3. Group III includes HbSAG12H5–12 and RcSAG12H4–5. Group IV includes HbSAG12H13–17, JcSAG12H7–8 and RcSAG12H6–8, which can be further divided into two subgroups (IVa and IVb) (**Fig 1**).

Obviously, a high number of genes were grouped in pairs, which is consistent with the homologous analysis above. *HbSAG12H2* and *3* were characterized by same-direction neighbors on scaffold0696 and can be defined as tandem duplications. In contrast, *HbSAG12H4* from scaffold1445, exhibits the high identity of 89.2% or 89.6% with *HbSAG12H2* and *3*, respectively, and is more likely to be resulted from the WGD. *HbSAG12H5–9* from scaffold0048, *HbSAG12H10–11* from scaffold2360 can be defined as tandem duplications, whereas their parental loci and *HbSAG12H12* from scaffold0420 are more likely to be resulted from the WGD. Interesting, *HbSAG12H13* and *14* (with 78.2% identity) from scaffold0247, *HbSAG12H15* and *16* (with 80.0% identity) from scaffold0583, were found to be tandem distributed on same scaffolds. However, according to the phylogenetic analysis, they were grouped into IVa or IVb, respectively, just like tandem duplications *JcSAG12H7* and *8*. The cluster result was further supported by the homologous analysis: *HbSAG12H13* and *14* exhibit relatively higher identity with *HbSAG12H16* (90.3%) or *HbSAG12H15* (93.0%), respectively; *HbSAG12H14* and *15* also show the high identity of 92.0% or 95.4% with *HbSAG12H17* from scaffold1086, respectively. Thereby, scaffold0247 and scaffold0583 are more likely to be resulted from the WGD, and the tandem duplications *HbSAG12H13*/*14* or *HbSAG12H15*/*16* are promised to be appeared before the divergence of rubber tree and physic nut. However, whether *HbSAG12H17* is a proximal duplication of *HbSAG12H15* still needs to be confirmed, since the 7,453 assembled scaffolds have not been anchored to the chromosomes yet [38].

### Sequence feathers of HbSAG12Hs

**Fig 2:**
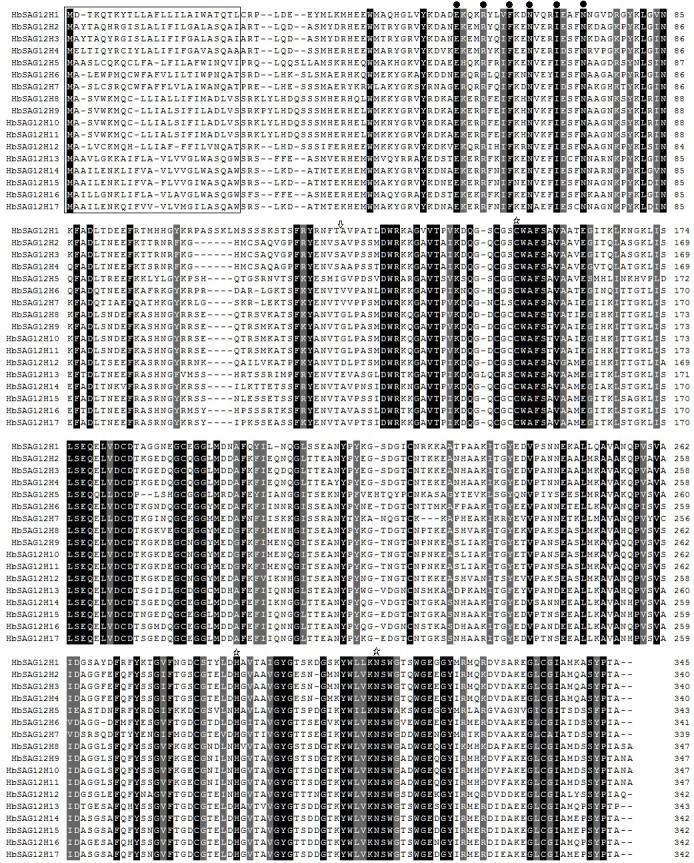
Alignment of precursor proteins of HbSAG12Hs. Sequence alignment was performed using MUSCLE and the alignment was displayed using Boxshade. Black shading shows identical amino acids, whereas light gray shading shows similar amino acids. The numbers indicate the positions of the amino acids within individual proteins. The consensus ERFNIN motif is marked with black dots. The conserved catalytic triad (Cys, His and Asn) is marked with asterisks. The predicted signal peptide is boxed. The putative cleavage site to generate the mature enzyme is marked with a down arrow, which is predicted based on sequence alignment with IbSPG31.

Sequence analysis showed that the 17 deduced HbSAG12H proteins consist of 339 to 347 amino acids with the theoretical MW ranging from 37352.7 to 38578.8 Da, which is consistent with AtSAG12, JcSAG12Hs, RcSAG12Hs and other reported SAG12 orthologues. The *p*I value of HbSAG12Hs ranges from 4.65 to 9.10. Although about 47.06% out of 17 HbSAG12Hs were predicted to be basic as AtSAG12, BnSAG12-2 and NtSAG12, the remainings are acid as BnSAG12-1, IbSPG31, Ghccyp and NtCP1. All HbSAG12Hs were predicted to harbor the GRAVY value (from -0.485 to -0.248) of less than 0, indicating their hydrophilic feather. As shown in **Fig 2**, all HbSAG12Hs contain the conserved catalytic triad (Cys-His-Asn), and a conserved ERFNIN motif with the exception of the R/H mutation in HbSAG12H6. All HbSAG12Hs were also predicted to harbor a hydrophobic signal peptide at the N-terminus (**Fig 2 and Table 1**).

**Fig 3:**
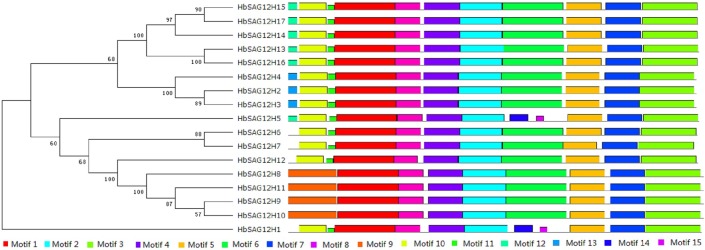
Structural and phylogenetic analysis of HbSAG12H proteins. The unrooted phylogenetic tree resulting from all the HbSAG12H proteins is shown on the left. The distribution of conserved motifs among the HbSAG12H proteins is shown on the right. Different motifs are represented by different color blocks as indicated at the bottom of the figure. The same color in different proteins indicates the same group or motif.

To learn more about the diversity of motif compositions among different HbSAG12Hs, a phylogenetic tree from 17 HbSAG12Hs was constructed and the motifs in protein sequences were predicted using MEME (**Fig 3**). Among the 15 identified motifs, motifs 1–5, 7 and 8 are broadly distributed. Motif 1 includes the ERFNIN consensus sequence and is characterized as the Inhibitor_I29 (Pfam accession number PF08246) which provides the core structure of the autoinhibitory prodomain. Motif 2 includes the Cys active site. Motif 3 includes the Asn active site. Motif 4 includes the putative cleavage site to generate the mature enzyme. Motif 7 includes the His active site. Motif 14 identified in HbSAG12H1 and 5 as well as motif 6 found in other HbSAG12Hs includes the GCE/Q/G/K/N/DGG consensus sequence, where the Cys residue is involved in the formation of a disulphide bridge. Motifs 9, 10, 12 and 13 belong to the predicted signal peptide. In contrast, little is known for other motifs: motifs 5 and 8 were found in all HbSAG12Hs; motif 11 was found in most HbSAG12Hs excluding HbSAG12H8–11; motif 15 was limited to HbSAG12H1 and 2 (**Fig 3 and Table 4**).

**Table 4:** Motif sequences of 17 HbSAG12H proteins identified by MEME tools.

Motif	E-value	Sites	Width	Best possible match
Motif 1	1.3e-534	17	50	NFITPPGTSNINANQPQRRRNWTREGEILPELLTQTFKRSLRLGVLPFLR
Motif 2	3.5e-441	17	36	QCGSCWAFSSVGALEGQLKKKTGKLLNLSPQNLVDC
Motif 3	3.1e-555	17	46	TGSPPYWIVRNSWGSSWGVDGYAHVKMGAPLLCILPAKPVSLSPEG
Motif 4	1.1e-326	17	29	MFPVAVNISIPASLDWREKGYVTPVKNQG
Motif 5	1.90E-307	17	29	INVKIALCVTDEASLVRLLAKQLVYVLVA
Motif 6	1.7e-447	15	50	SWPQGNEGCNGGLMDYAFQYVKDNGGLDSEKSYPYSGKDETCHYRPQDSA
Motif 7	3.50E-304	17	29	SITAVATCVFSSPPGRRLHHAVTPHGKGG
Motif 8	7.20E-133	17	21	LTNEESRARYDHWRRSQVSMP
Motif 9	2.80E-94	4	41	LLSIRSLLLLLNLPHVMLLPEVVNMLALLLEDWTALMHLRR
Motif 10	5.70E-79	13	23	AFLLPLVVALPKTLAIPEKLQEA
Motif 11	1.10E-15	13	6	MTERHE
Motif 12	6.80E-03	6	8	MSAILEDK
Motif 13	5.30E-01	3	8	MQLTAQLR
Motif 14	9.10E+01	2	15	EGCNGGLMDYAFQYV
Motif 15	2.50E+03	2	6	SEKNFP

### Expression profiles of *HbSAG12H* genes

**Fig 4:**
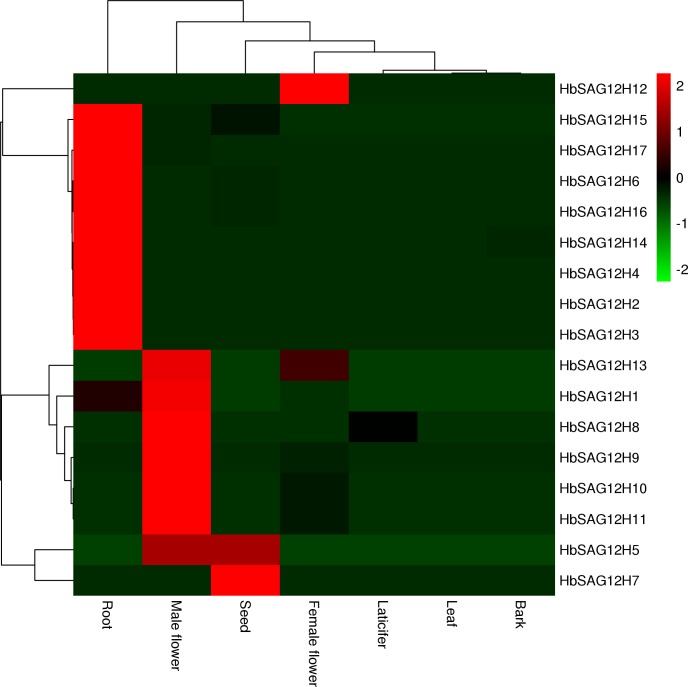
Tissue-specific expression profiles of 17 *HbSAG12H* genes. Color scale represents FPKM normalized log_10_ transformed counts where green indicates low expression and red indicates high expression.

As shown in **Fig 4**, transcriptome profiling showed that most *HbSAG12H* genes were not or lowly expressed in examined tissues except for root and male flower. Among 10 genes detected in male flower, the transcripts of *HbSAG12H11*, *HbSAG12H10*, *HbSAG12H9* and *HbSAG12H1* were relatively abundant. The high abundance of *AtSAG12* and four *RcSAG12Hs* (i.e. *RcSAG12H5*, *RcSAG12H4*, *RcSAG12H1* and *RcSAG12H3*) in flower was also observed. In root, the more abundant transcripts include *HbSAG12H4*, *HbSAG12H3*, *HbSAG12H17*, *HbSAG12H2*, *HbSAG12H15*, *HbSAG12H14*, *HbSAG12H16* and *HbSAG12H6*, and their cDNAs can indeed be amplified using roots as the template (data not shown). In addition, the abundant *HbSAG12H7* seems to be a seed-specific gene since its transcript was not detected in other examined tissues.

**Fig 5:**
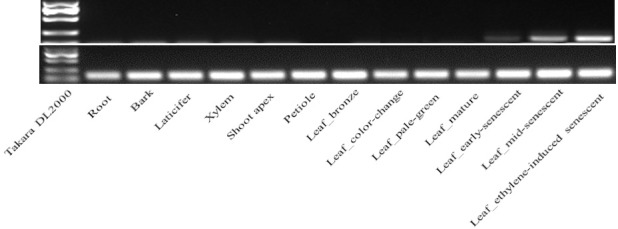
Semi-quantitative RT-PCR analysis of *HbSAG12H1*.

Moreover, 17 primer pairs were also used to amplify *HbSAG12H* genes expressed in senescent leaves. Interesting, the only amplified gene was confirmed to be *HbSAG12H1*. Thereby, subsequent semi-quantitative RT-PCR analysis was focused on *HbSAG12H1*. Except for flower and seed, all profiled tissues as well as shoot apex, petiole, xylem and leaf of six more developmental stages, were examined. As shown in **Fig 5**, the expression of *HbSAG12H1* was only found in senescent leaves, i.e., lowly in early-senescent leaves, moderately in mid-senescent leaves, and highly in ET-induced senescent leaves. The result is consistent with its transcriptome profiles in laticifers, barks and mature leaves. In contrast, no detected PCR product of this gene in roots is more likely to be due to its low expression in this specific tissue, which usually results in no visible signal by electrophoresis. And RNA-seq (RNA sequencing) has been proved to be the most sensitive technology to determine gene expression [60].

## Discussion

Gene duplication is a major mechanism for acquiring new genes, which may be resulted from single gene duplications such as local (tandem or proximal), dispersed and transposed duplications, or large-scale duplications such as WGDs and segmental duplications [61]. WGDs are widespread in plants. For example, one so-called γ whole-genome triplication event was shown to occur in all core eudicot plants including *Arabidopsis*, rubber tree, castor and physic nut [62]. Moreover, it is well established that *Arabidopsis* underwent two recent doubling events [63,64]. Nevertheless, *Arabidopsis SAG12* was found to exist as a single copy gene [65]. The present study performed the first genome-wide identification of *SAG12*-like genes in rubber tree, and an unexpected high number of 17 orthologues were found. Thereby, to reveal their expansion and evolutionary pattern is particularly interesting.

In Euphorbiaceae, the genome sequences of castor and physic nut are also available. Unlike rubber tree, both castor and physic nut didn’t undergo any recent WGD [38,43–45]. Thereby, the identification of SAG12 orthologues in these two plants may provide insights into the evolutionary pattern of *HbSAG12H* genes. The genome-wide survey indicated that both castor and physic nut contain 8 SAG12 orthologues, and the number is two-folds smaller than that in rubber tree. Without any exception, all *SAG12H* genes in rubber tree, castor and physic nut contain a single intron as observed in sweet potato [19], but not in *Arabidopsis* and rapeseed that harbor two introns [11,18].

The phylogenetic analysis was further performed to investigate the evolutionary relationships of HbSAG12Hs, which divided the tested SAG12 orthologues into four groups. Interesting, rubber tree harbors at least one orthologue in each group. In contrast, no orthologue can be found in Group I and III for castor or physic nut, respectively. This means that the diversification of SAG12 orthologues into four groups can date back to the ancestral Euphorbiaceae plant, and group-specific gene loss has occurred in both castor and physic nut. In fact, all group members can be found in poplar (*Populus trichocarpa*), and members of Group I-III in papaya (*Carica papaya*) [66,67]. Since papaya underwent no recent WGD and stands very close to *Arabidopsis*, the unique *AtSAG12* is more likely to be resulted from massive gene loss and chromosomal rearrangement after WGDs [64].

According to the phylogenetic analysis, a high number of gene pairs were identified, i.e., 3 in castor, 3 in physic nut and 5 in rubber tree. The duplication mechanism was investigated based on their locations. In castor and physic nut, except for *JcSAG12H1*/*2* that can be defined as proximal duplications, other gene pairs were all derived from tandem duplications, supporting its major role in the expansion of SAG12 orthologues. In contrast, a role of WGD is also involved in rubber tree: *HbSAG12H2/3*, *HbSAG12H5–9*, *HbSAG12H10/11*, *HbSAG12H13/14* and *HbSAG12H15/16* can be defined as tandem duplications, while *HbSAG12H4* or *HbSAG12H2/3*, *HbSAG12H12* or *HbSAG12H10/11*, *HbSAG12H13/14* or *HbSAG12H15/16* are more likely to be resulted from the WGD.

Like AtSAG12 and other reported papain-like cysteine proteases, sequence analysis indicated that HbSAG12Hs are synthesized as inactive prepropeptides, consisting of an N-terminal signal sequence followed by an autoinhibitory prodomain, and the mature enzyme (i.e. the Peptidase_C1 domain under the Pfam accession number of PF00112) at the C-terminus. And the mature, active enzyme is promised to be resulted from proteolysis cleaving off the pre and pro domains [68]. The putative cysteine protease activity of HbSAG12Hs is supported by the presence of the conserved catalytic triad Cys-His-Asn, which was shown to be essential for the cysteine protease activity [69,70]. Moreover, the expression of *HbSAG12H* genes were all supported by available RNA sequencing reads, suggesting that they have function in rubber tree. Nevertheless, transcriptome profiling indicated that the expression level of most *HbSAG12H* genes was considerably low in most examined tissues, and the result was further confirmed by the RT-PCR analysis. When using roots as the PCR template, 8 abundant genes can be successfully amplified, whereas no amplification can be observed for the less abundant *HbSAG12H1* and other *HbSAG12H* genes (data not shown). In fact, *HbSAG12H1* presents the only gene that can be amplified from senescent leaves (**Fig 5**). The senescence-specific expression of *HbSAG12H1* in leaves was further confirmed by the semi-quantitative RT-PCR analysis. Among 6 stages of developmental leaves (i.e. bronze, color-change, pale-green, mature, early- and mid-senescent) examined, the expression of *HbSAG12H1* was limited to senescent leaves and the transcript level gradually increased during leaf senescing. In addition, the transcripts of *HbSAG12H1* increased about 10 folds in ET-induced senescent leaves than that in mid-senescent leaves (**Fig 5**). Since mid-senescent and ET-induced senescent leaves contain the similar chlorophyll content (i.e. 45–55% relative to mature leaves), the expression of *HbSAG12H1* is more likely to be induced by ET, which is consistent with the presence of several putative ET-responsive elements in its 2000-bp promoter region (data not shown). Similar expression patterns were also reported for other SAG12 orthologues [18–22]. For examples, among 5 tissues (i.e. leaf, flower, stem, root and tuber) tested, the transcripts of *SPG31* were detected only in senescent leaves, and the transcript level can be highly induced after treatment of mature leaves with ET for 3 days [19]; both *BnSAG12-1* and *BnSAG12-2* transcripts were detected only in senescent cotyledons and leaves [18]. Although the mechanism of the transcript abundance of *HbSAG12H* genes in male flowers and roots is yet to be elucidated, group I and III members tend to be expressed in male flowers, whereas group II and IV members prefer to be expressed in roots.

## Conclusions

In this study, survey of the rubber tree genome resulted in 17 SAG12 orthologues, and the gene number is considerably larger than 8 in both castor and physic nut, another two Euphorbiaceae plants. These orthologues can be divided into four groups based on the phylogenetic analysis. Genome-wide comparative analysis indicated that the diversification of SAG12 orthologues can date back to the ancestor of core eudicot plants: the unique *AtSAG12* in *Arabidopsis* is resulted from gene loss; the duplicated SAG12 orthologues in castor and physic nut were mainly resulted from tandem duplications; whereas the duplicated *HbSAG12H* genes were derived from tandem duplications as well as the recent WGD. Furthermore, *HbSAG12H1* exhibits a strictly senescence-associated expression pattern in rubber tree leaves, and can be used as a marker gene for the study of senescence mechanism in *Hevea*.

## Supporting information

S1 File PCR conditions used in this study. (PDF)

S2 File The gene model for *JcSAG12H8*. (PDF)

S3 File The gene model for *JcSAG12H1*. (PDF)

S4 File The gene model for *JcSAG12H2*. (PDF)

S5 File The gene model for *JcSAG12H7*. (PDF)

S1 Table PCR primers used for gene cloning in this study. (XLSX)
