# Supplementary material for: Survey of the rubber tree genome reveals a high number of cysteine protease-encoding genes homologous to Arabidopsis SAG12
Source: PLoS One. 2017 Feb 6;12(2):e0171725. doi: 10.1371/journal.pone.0171725 (PMC5293227; doi:10.1371/journal.pone.0171725)
Supplement: S1 File — (PDF) [file pone.0171725.s001.pdf]

S1 File. PCR conditions used in this study.

A． For gene cloning

The 50 mL reaction mixture contains 10 μl 5×Takara PrimeSTAR Buffer (Mg^{2+} Plus), 10 μM dNTP Mixture, 400 nM of each primer, 1.25 u Takara PrimeSTAR HS DNA Polymerase and about 30 ng cDNA.

The PCR procedure is as follows: 3 min at 94℃ for initial denaturation, followed by 35 cycles of denaturation 30 s at 94 ℃, 1 min at 55 ℃.

B． For semi-quantitative RT-PCR

The 50 mL reaction mixture contains 5 μl 10×PCR Buffer (Mg^{2+} Plus), 10 μM dNTP Mixture, 400 nM of each primer, 1.25 u Takara Taq and about 30 ng cDNA.

The PCR procedure is as follows: 3 min at 94℃ for initial denaturation, followed by 18 (for *Hb18S rRNA*) or 35 (for *HbSAG12H1*) cycles of denaturation 30 s at 94 ℃, 15 s at 55 ℃.
