# Supplementary material for: Survey of the rubber tree genome reveals a high number of cysteine protease-encoding genes homologous to Arabidopsis SAG12
Source: PLoS One. 2017 Feb 6;12(2):e0171725. doi: 10.1371/journal.pone.0171725 (PMC5293227; doi:10.1371/journal.pone.0171725)
Supplement: S2 File — (PDF) [file pone.0171725.s002.pdf]

S2 File. The gene model for *JcSAG12H8*. The coding region is marked with uppercase letters, above which is its deduced amino acids. The transcribed untranslated regions, including 5’ UTR, intron and 3’ UTR sequences, are marked with lowercase letters. The start and stop codons are marked with bold letters.

1 gaaactgaattttggttgtccatatcatacacaactaacgggattaactcaacataagac

61 tgcaatttcagacatctcttgtaaggaagccaatttatatgttctataaatacatgacag

121 ctctttaaggactagtgtgccaaaaatacacatatcctacattatatactctctagtctt

1 M A S I L E N K L V F V T

181 tacttcctcctccaccaagaaga**ATG**GCTTCAATTCTTGAAAACAAACTGGTCTTTGTGA

14 V L V L G L F A S Q A F A R P L Q N E T

241 CAGTGTTGGTGCTGGGGCTCTTTGCATCTCAAGCCTTTGCACGTCCTCTACAAAATGAAA

34 M K E R H E M W M A K Y G R V Y K D S A

301 CCATGAAGGAGAGGCATGAAATGTGGATGGCTAAATATGGACGTGTTTATAAAGACAGTG

54 E K E K R F N I F K N N V E F I E S F N

361 CAGAGAAGGAGAAACGCTTTAACATATTCAAGAATAATGTGGAGTTCATTGAATCTTTTA

74 K D G N K L Y K L D I N G F A D L S N E

421 ATAAGGATGGGAATAAGCTTTACAAGCTAGATATCAATGGATTTGCAGACTTAAGTAATG

94 E F K A S R N G Y K R S S I A K S S E T

481 AAGAGTTTAAGGCCTCTAGAAATGGATATAAAAGATCTTCTATTGCTAAGTCATCTGAGA

114 L S F K Y E N V T A V P T S M Y W R N K

541 CGCTGTCGTTTAAGTATGAAAATGTCACCGCTGTCCCAACTTCCATGTACTGGAGGAACA

134 G A V T P I K D Q G Q C G

601 AAGGAGCTGTTACTCCCATCAAGGACCAAGGCCAATGCGgtaaggaacacaatgtttctt

661 atttttcttctactatgtactagctatagtaaaattaatctaatatatgtatttcccttt

147 C C W A F S A V A A M E G I

721 tggaacacacaaatgtagGATGTTGCTGGGCATTTTCTGCTGTGGCTGCCATGGAAGGAA

161 T K L S T G K L I S L S E Q E L V D C D

781 TTACAAAGCTCTCAACAGGAAAATTGATTTCTCTTTCTGAGCAAGAACTTGTTGATTGTG

181 T S G E D Q G C E G G L M D D A F E F I

841 ACACAAGTGGAGAAGATCAAGGCTGCGAAGGGGGTCTTATGGATGATGCATTTGAATTCA

201 K K N G G L T T E A N Y P Y Q G T D D S

901 TAAAAAAAAATGGAGGACTAACAACTGAAGCCAATTACCCTTACCAAGGAACTGATGATT

221 C N K R K A V D H A A K L T G Y E D V P

961 CTTGCAATAAAAGAAAGGCAGTTGATCACGCAGCAAAGCTTACTGGCTATGAAGATGTGC

241 A N S E D A L L K A V A N Q P V S V A I

1021 CTGCTAATAGTGAAGATGCCTTATTGAAGGCAGTAGCCAACCAACCAGTCTCTGTAGCCA

261 D A S G S A F Q F Y S G G V F T G D C G

1081 TTGATGCAAGTGGCTCTGCTTTCCAGTTCTACTCAGGTGGAGTATTTACAGGAGATTGCG

281 T E L D H G V T A V G Y G T S S D G T K

1141 GAACTGAACTCGACCATGGAGTTACTGCAGTTGGGTATGGCACAAGCAGTGATGGAACCA

301 Y W L V K N S W G T S W G E N G Y I R M

1201 AGTATTGGTTAGTGAAGAACTCATGGGGAACTTCTTGGGGTGAAAATGGATACATTAGAA

321 E R D I D A S E G L C G I A M E P S Y P

1261 TGGAAAGAGATATTGATGCTAGCGAAGGTCTTTGTGGAATTGCGATGGAACCTTCTTACC

341 T A *

1321 CAACTGCA**TAA**ttaaaattaagtggcagaaaagaaaatatgggtcaatcttaaaatataa

1381 atacattgtccaaaa
