# Supplementary material for: Survey of the rubber tree genome reveals a high number of cysteine protease-encoding genes homologous to Arabidopsis SAG12
Source: PLoS One. 2017 Feb 6;12(2):e0171725. doi: 10.1371/journal.pone.0171725 (PMC5293227; doi:10.1371/journal.pone.0171725)
Supplement: S3 File — (PDF) [file pone.0171725.s003.pdf]

S3 File. The gene model for *JcSAG12H1*. The coding region is marked with uppercase letters, above which is its deduced amino acids. The transcribed untranslated regions, including 5' UTR, intron and 3' UTR sequences, are marked with lowercase letters. The start and stop codons are marked with bold letters.

1 M T K K Q S

1 caaagattccttctgtatagttatagatactctcttgatact**ATG**ACTAAAAAACAAAGC

7 K S I F L V F V L N I L T I W A T H T V

61 AAATCCATATTTCTGGTATTTGTGTTGAACATATTAACCATATGGGCTACACATACGGTT

27 C R P L N E E Y M L K R H E E W R A Q H

121 TGTCGTCCTCTTAACGAAGAATACATGTTAAAGAGGCATGAAGAATGGAGAGCCCAACAT

47 G R V Y K D T A E K Q K K Y L V F K D N

181 GGACGTGTCTACAAAGACACAGCAGAGAAACAGAAAAAATACCTGGTTTTTAAGGACAAC

67 L E R I E S F N N G V D R G Y K L G L N

241 CTTGAACGTATTGAATCCTTTAACAATGGTGTGGACCGTGGATACAAGCTAGGACTCAAC

87 K F A D L T D E E F Q A M H L G Y K S P

301 AAATTTGCAGACTTAACAGATGAGGAATTTCAGGCCATGCACCTTGGTTACAAGAGCCCA

107 P S K L M S T S K S R S F R Y R N V T S

361 CCCTCCAAATTAATGTCCACTTCAAAGTCCAGATCCTTTAGGTACAGAAATGTAACTTCT

127 V P T T I D W R K A G A V T P V K D Q G

421 GTGCCAACTACTATAGATTGGAGAAAGGCCGGTGCTGTGACCCCTGTCAAAGATCAAGGC

147 S C G

481 TCCTGCGgtgagtgctgatataatcttaatatgtaaggcagaagtagacctaggatttag

541 aaccttaacttgtcatactatgagacagactcaaccactgtcctattgctaaataaatac

601 acatgaaattgaacaatgattttgatcttaaaaatcatgctattgatgcataacagaaaa

661 gagcaatccccgcaccacaaatgaaaattcgtttctaataaatcatttttaatgtttaa

150 S C W A F S A V A A M E G I T K L

721 tgcatagGAAGTTGCTGGGCATTCTCAGCAGTGGCAGCAATGGAAGGGATCACAAAACTC

167 K T G N L I S L S E Q E L V D C D V A G

781 AAAACTGGCAATTTAATATCTTTATCAGAGCAAGAGCTCGTAGATTGCGACGTAGCAGGT

187 E D Y G C D G G F M D T A F Q Y I L K N

841 GAGGATTATGGTTGTGACGGAGGTTTCATGGACACTGCTTTCCAATATATCCTAAAAAAT

207 G G L T S E A N Y P Y Q G E D G I C S K

901 GGAGGTCTCACGAGTGAGGCTAATTACCCCTACCAAGGAGAAGATGGCATCTGCAGCAAG

227 K K T A T S M A K I T G Y E D V P S N S

961 AAGAAGACAGCAACTTCTATGGCTAAAATAACTGGATATGAAGATGTGCCATCTAACAGT

247 E K A L L Q A V A N Q P V S V A I D A S

1021 GAAAAGGCTCTCTTGCAAGCTGTGGCAAACCAACCAGTTTCTGTTGCTATTGATGCTAGT

267 G Y D F R F Y S S G V F K G D C P T D L

1081 GGGTATGACTTCAGATTTTACTCTTCTGGTGTTTTTAAAGGGGACTGTCCTACCGATCTC

287 N H A V T V I G Y G S S S D G T K Y W L

1141 AACCATGCTGTTACTGTAATTGGGTATGGTAGTAGCAGTGATGGTACTAAGTATTGGTTG

307 L K N S W G T G W G E N G Y M R M Q R E

1201 CTAAAGAATTCATGGGGCACCGGTTGGGGTGAGAATGGGTATATGAGGATGCAAAGGGAA

327 I S A N E G L C G I A M K A S Y P T A *

1261 ATTAGTGCAAACGAAGGCCTCTGTGGCATTGCCATGAAAGCTTCGTATCCAACTGCT**TGA**

1321 aattgaacaaggagaagcataatggcaataaatcctatttgatgtgttagtatcaggaaa

1381 tttataggctgtaaaatgttctgctatctgtgctcattctatctttcatgcagttgtata

1441 atttcagcaatatgtgtttactatccatgcataaaggttcatttattttagctatcaagt

1501 accaac
