# Supplementary material for: Survey of the rubber tree genome reveals a high number of cysteine protease-encoding genes homologous to Arabidopsis SAG12
Source: PLoS One. 2017 Feb 6;12(2):e0171725. doi: 10.1371/journal.pone.0171725 (PMC5293227; doi:10.1371/journal.pone.0171725)
Supplement: S4 File — (PDF) [file pone.0171725.s004.pdf]

S4 File. The gene model for *JcSAG12H2*. The coding region is marked with uppercase letters, above which is its deduced amino acids. The transcribed untranslated regions, including 5’ UTR, intron and 3’ UTR sequences, are marked with lowercase letters. The start and stop codons are marked with bold letters.

```
  1                                                    M  T  K  K  Q  S
  1 caaagattccttctttatagttatagatactctcttgatactATGACTAAAAAACAAAGC
  7 K  S  I  F  L  V  F  V  L  N  I  L  T  I  W  A  T  H  T  V
 61 AAATCCATATTTCTGGTATTTGTGTTGAACATATTAACCATATGGGCTACACATACGGTT
 27 C  R  P  L  N  E  E  Y  M  L  K  R  H  E  E  W  R  A  Q  H
121 TGTCGTCCTCTTAACGAAGAATACATGTTAAAGAGGCATGAAGAATGGAGAGCCCAACAT
 47 G  R  V  Y  K  D  T  A  E  K  Q  K  K  Y  L  V  F  K  D  N
181 GGACGTGTCTACAAAGACACAGCAGAGAAACAGAAAAAATACCTGGTTTTTAAGGACAAC
 67 L  E  R  I  E  S  F  N  N  G  V  D  R  G  Y  K  L  G  L  N
241 CTTGAACGTATTGAATCCTTTAACAATGGTGTGGACCGTGGATACAAGCTAGGACTCAAC
 87 K  F  A  D  L  T  D  E  E  F  R  A  M  H  L  G  Y  K  S  L
301 AAATTTGCAGACTTAACAGATGAGGAATTTCGGGCTATGCACCTTGGTTACAAGAGCCTA
107 P  S  K  L  M  A  T  S  K  S  R  S  F  R  Y  R  N  V  T  S
361 CCCTCCAAATTAATGGCCACTTCAAAGTCCAGATCCTTTAGGTACAGAAATGTAACTTCC
127 V  P  T  T  I  D  W  R  K  A  G  A  V  T  L  V  K  D  Q  G
421 GTGCCAACTACTATAGATTGGAGAAAGGCTGGTGCTGTGACCCTTGTCAAAGATCAAGGC
147 S  C  G
481 TCCTGCGgtaagtgctgatataatcttaatatataaggcagaagtagacctaggatttag
541 aaccttaacttgtcatactatgagacagactcaaccattgtcctattgctaaataaatac
601 acataaaaacaatgattttgatcttaaaaatcatgctattgatgcataacagaaaagagc
661 aatccccgtaccagaaatgaaaattcatttctaataaatcatttttaatgtttaatgca
150    C  C  W  A  F  S  A  V  A  A  M  E  G  I  T  K  L  K  T
721 tagGATGTTGCTGGGCATTCTCAGCAGTGGCAGCAATGGAAGGAATCACAAAACTTAAAA
169  G  K  L  I  S  L  S  E  Q  E  L  V  D  C  D  I  A  G  E  D
781 CTGGCAAGTTAATATCTTTATCAGAGCAAGAGCTCGTAGATTGTGACATAGCAGGTGAGG
```

189 Y G C D G G F I D T A F Q Y I L K N G G

841 ATTATGGTTGTGACGGAGGTTTCATAGACACTGCTTTCCAATATATCCTAAAAAATGGAG

209 L T S E A N Y P Y Q G E D G I C S K K K

901 GTCTCACGAGTGAGGCTAATTACCCCTACCAAGGAGAAGATGGCATCTGCAGCAAGAAGA

229 T A T S T A K I T G Y E D V P S N S E K

961 AGACAGCAACTTCTACGGCTAAGATAACTGGATATGAAGATGTGCCATCTAACAGTGAAA

249 A L L Q A V A N Q P V S V A I D A S G Y

1021 AGGCTCTCTTGCAAGCTGTGGCAAACCAACCAGTTTCTGTTGCTATTGATGCTAGTGGGT

269 D F R F Y S S G V F Q G D C T T Y L N H

1081 ATGACTTCAGATTTTACTCTTCTGGTGTCTTTCAAGGGGACTGTACTACCTATCTAAACC

289 A V T V I G Y G S S S Y G T K Y W L L K

1141 ATGCTGTTACTGTAATTGGGTATGGTAGTAGCAGTTATGGTACTAAGTATTGGTTGCTAA

309 N S W G T G W G E N G Y M R M Q R E I S

1201 AGAATTCATGGGGCACCGGTTGGGGTGAGAATGGGTATATGAGGATGCAAAGGGAAATTA

329 A N E G L C G I A M K A S Y P T A *

1261 GTGCAAATGAAGGCCTCTGTGGCATTGCCATGAAAGCTTCGTATCCAACTGCT**TGA**aatt

1321 gaacaggagaagcataatggcaataaatcctatttgatgtgttagtatcaggaaatttat

1381 aggctgcaaaatgttctgctatctgtgctcattctatctttcatgcagttgtataatttc

1441 agcaatatgtgtttaatatccatgtataaaggttcatttattttagctatcaagtaccaa

1501 t
