# Supplementary material for: Survey of the rubber tree genome reveals a high number of cysteine protease-encoding genes homologous to Arabidopsis SAG12
Source: PLoS One. 2017 Feb 6;12(2):e0171725. doi: 10.1371/journal.pone.0171725 (PMC5293227; doi:10.1371/journal.pone.0171725)
Supplement: S5 File — (PDF) [file pone.0171725.s005.pdf]

S5 File. The gene model for *JcSAG12H7*. The coding region is marked with uppercase letters, above which is its deduced amino acids. The intron sequences are marked with lowercase letters. The start and stop codons are marked with bold letters.

1 M A V V V L L E N K L I F V A V L V M G

1 **ATG**GCTGTAGTAGTACTACTTGAAAACAAACTAATCTTTGTTGCAGTGTTAGTGATGGGG

21 L C A C Q G W S R S L Y E A S L E E R H

61 TTGTGTGCTTGCCAAGGCTGGTCACGTTCCCTCTATGAAGCTTCGTTGGAGGAGAGGCAT

41 E I W M A Q H E R V Y K D A T E K E M R

121 GAAATTTGGATGGCCCAACATGAACGTGTTTATAAAGACGCGACAGAAAAGGAGATGCGC

61 F Q I F R E N V K F I E S F N K A G N K

181 TTTCAGATATTCAGGGAGAATGTGAAATTCATTGAATCTTTCAACAAGGCTGGGAATAAG

81 S Y K L G I N A F T D L T N K E F R A S

241 TCTTATAAGCTAGGGATTAATGCATTTACAGACCTCACTAATAAGGAATTTCGTGCATCG

101 R N G Y K R S S Y S S S S G T N S F K Y

301 CGAAATGGATACAAGAGGTCTTCTTATTCAAGCTCATCAGGAACAAACTCATTCAAATAT

121 E N V T T I P S S L D W R S K G V V T P

361 GAAAATGTCACTACAATTCCATCTAGCTTGGATTGGAGAAGCAAAGGAGTCGTTACACCC

141 I K D Q G Q C G

421 ATCAAAGACCAAGGTCAATGTGgtaagtaacttggacttcatgtaaacaagtcagggttt

481 caaaatcctgatagtccatccctttaagaattgtgataaaagaaaaaaataggaaaagga

149 C C W

541 aaaaagaaagaaacctcttaacattatatgaattgcatgcaaaaatgcagGATGTTGTTG

152 A F S A V A A M E G I T K L S T E K L I

601 GGCATTCTCTGCGGTGGCAGCTATGGAAGGGATCACAAAACTCTCAACTGAGAAATTGAT

172 S L S E Q E L I D C D T S G I D Q G C E

661 CTCCCTTTCTGAGCAAGAATTGATTGACTGTGATACAAGTGGAATAGACCAAGGCTGTGA

192 G G L M D D A F E F I I Q N N G L T T E

721 GGGTGGTCTTATGGATGATGCCTTTGAATTCATTATTCAAAATAATGGCCTTACGACTGA

212 A N Y P Y Q A E D G T C N T E K A A N H

781 AGCAAATTATCCTTACCAGGCAGAGGATGGAACTTGCAACACCGAGAAAGCAGCAAATCA

232 A A T I T S Y E D V P E N N E E A L R M

841 TGCTGCCACAATCACAAGTTATGAAGATGTGCCAGAAAACAATGAGGAGGCCTTACGCAT

252 A V A N Q P V S V A I D A G E S A F Q H

901 GGCAGTGGCAAACCAACCAGTTTCTGTTGCCATTGATGCTGGTGAATCTGCCTTCCAACA

272 Y S S G I F T G D C G T E L D H G V T V

961 CTACTCGAGTGGAATATTTACTGGAGATTGTGGAACTGAATTAGACCACGGTGTCACTGT

292 V G Y G T S D D G T K Y W L V K N S W G

1021 AGTCGGTTATGGGACAAGTGATGATGGGACTAAGTATTGGTTAGTAAAGAACTCATGGGG

312 T S W G E D G Y I R M Q R D I D A K E G

1081 AACCAGCTGGGGTGAGGATGGATACATTAGAATGCAAAGAGACATTGATGCTAAAGAAGG

332 L C G I A M Q P S Y P T A *

1141 CCTATGTGGAATTGCCATGCAGCCTTCCTATCCAACTGCA**TAA**
